# Efficient double-quenching of electrochemiluminescence from CdS:Eu QDs by hemin-graphene-Au nanorods ternary composite for ultrasensitive immunoassay

**DOI:** 10.1038/srep30577

**Published:** 2016-07-27

**Authors:** Jing Liu, Meirong Cui, Hong Zhou, Shusheng Zhang

**Affiliations:** 1 Shandong Provincial Key Laboratory of Detection Technology for Tumor Markers, College of Chemistry and Chemical Engineering, Linyi University, Linyi 276005, P. R. China; 2 Collaborative Innovation Center of Functionalized Probes for Chemical Imaging in Universities of Shandong, Shandong Normal University, Jinan 250014, P. R. China

## Abstract

A novel ternary composite of hemin-graphene-Au nanorods (H-RGO-Au NRs) with high electrocatalytic activity was synthesized by a simple method. And this ternary composite was firstly used in construction of electrochemiluminescence (ECL) immunosensor due to its double-quenching effect of quantum dots (QDs). Based on the high electrocatalytic activity of ternary complexes for the reduction of H_2_O_2_ which acted as the coreactant of QDs-based ECL, as a result, the ECL intensity of QDs decreased. Besides, due to the ECL resonance energy transfer (ECL-RET) strategy between the large amount of Au nanorods (Au NRs) on the ternary composite surface and the CdS:Eu QDs, the ECL intensity of QDs was further quenched. Based on the double-quenching effect, a novel ultrasensitive ECL immunoassay method for detection of carcinoembryonic antigen (CEA) which is used as a model biomarker analyte was proposed. The designed immunoassay method showed a linear range from 0.01 pg mL^−1^ to 1.0 ng mL^−1^ with a detection limit of 0.01 pg mL^−1^. The method showing low detection limit, good stability and acceptable fabrication reproducibility, provided a new approach for ECL immunoassay sensing and significant prospect for practical application.

Sensitive and accurate detection of serum or tissue biomarkers becomes increasingly important in biomedical research, biodefense applications and early diagnosis of diseases^1^,^2^,^3^,^4^. Most current detecting methods such as radioimmunoassay^5^,^6^, fluorescence immunoassay^7^,^8^, enzyme-linked immunosorbent assay (ELISA)^9^,^10^, chemiluminescence immunoassay^11^,^12^,^13^,^14^, mass spectrometric immunoassay^15^, which have been used for clinical sample detections are faced with some key problems. For example, sensitivity and accuracy and most conventional methods are inefficient for some low-abundance determinand. Therefore, it is still urgently needed to develop simple and ultrasensitive methods to satisfy the requirements in clinical diagnosis.

Recently, electrochemiluminescent (ECL) immunoassay based on quantum dots (QDs) has been rapidly developed in this field because it showed intrinsic low background and excellent sensitivity^16^,^17^,^18^,^19^. Furthermore, owing to the similar nature between the QDs-based ECL emission and photoluminescent (PL), energy transfer has been considered for the design of new QDs-based ECL analytical methodologies^20^,^21^,^22^,^23^,^24^,^25^. In this strategy, perfect energy overlapped donor/acceptor pair is a key factor to obtain optimal ECL-ET efficiency. Then to find energy tunable materials which could be employed as potential donor and acceptor is very important and becomes a hot topic for ECL-ET.

Using consumption of a coreactant (for example H_2_O_2_) during enzymatic reactions on the immunosensor surface is one primary principle of sensitive QDs-based ECL methodology^26^,^27^,^28^. Rencent study showed that this approaches based on natural enzymes probes were faced with many problems including complicated preparation process of enzyme linked probe and the misgivings of denaturation which greatly limited its practical applications. Therefore, another coreactant consumption probe enzyme mimics with high electrocatalytic reactivity and convenient storage and treatment attracted increasing interests. Among these enzyme mimics, for the reason that graphene oxide (GO) provides large specific surface areas and other specific properties, it has been widely employed for construction of devices involving catalytic processes. With its rapid development, more and more soluble GO-based hybrid peroxidase mimetics were developed and applied due to its good catalytic activity^29^,^30^. However, rare attention has been paid to the applications of mentioned GO-based hybrid peroxidase mimetics in smart ECL sensing. A ternary complex H-RGO-Au NRs was first synthesized and reported during our previous work^31^ and recently we found that it showed excellent performance during ECL biosensing which has not been reported. Peroxidase-like activity of H-RGO-Au NRs could consume ECL coreactant (H_2_O_2_) during enzymatic reactions on the immunosensor surface, resulting ECL signal diminution. Furthermore, with the combination of our previous interesting discovery that ECL resonance energy transfer (ECL-RET) could generate between Au NRs and the CdS:Eu QDs^32^, the introduce of the ternary complex is expected to realize further ECL quenching with the abundant Au NRs on its surface.

Here, we first presented a novel ultrasensitive ECL immunoassay method based on the high electrocatalytic activity of ternary complexes hemin-reduced graphene oxide-Au nanorods (H-RGO-Au NRs) for the reduction of H_2_O_2_ as the coreactant of QDs-based ECL, and the ECL resonance energy transfer (ECL-RET) strategy between the abundant Au NRs on the graphene triple complex surface and the CdS:Eu QDs (Fig. 1). The designed immunoassay method showed a linear range from 0.01 pg mL^−1^ to 1.0 ng mL^−1^ with a detection limit of 0.01 pg mL^−1^. Compared former reported material^33^, this novel H-RGO-Au NRs showed many unique and interesting properties including large specific surface, with abundant Au NRs for ECL-RET and peroxidase-like activity, all of which were favorable conditions for ECL biosensor. The sensing platform provided a feasible way to detect different cancer biomarkers by changing antibody. Also, the method provided a new approach for ECL immunoassay sensing and significant prospect for practical application.

## Results and Discussion

### Characterization of the materials

**Figure 2:**
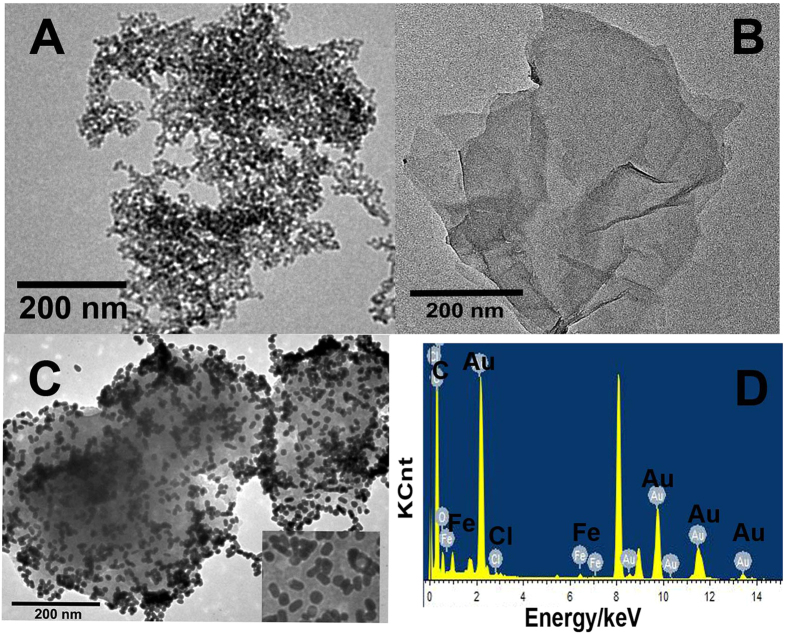
TEM images of CdS:Eu QDs (**A**), GO (**B**), H-RGO-Au NRs (**C**) and the EDX analysis of H-RGO-AuNRs (**D**). Insert of (**C**) shows the corresponding partly enlarged view of H-RGO-Au NRs.

TEM images of employed materials were shown in Fig. 2. Therein, Fig. 2A demonstrates the surface topography of doped CdS:Eu QDs with about 6 nm diameter. And for pure GO, a thin layer morphology was found in Fig. 2B. And after modification with hemin by heating a hemin and GO mixture with reflux in ammonia in the presence of hydrazine, the formed hemin-modified GO (H-RGO) was used as a substrate material for growth of Au NRs *in situ* through reduction of HAuCl_4_ by NaBH_4_ and ascorbic acid (AA). From Fig. 2C, we could find that Au NRs were successfully generated and grew on the surface of the H-RGO. The TEM image also showed that Au NRs were homogeneous with size of 20 × 10 nm and covered on the surface of H-RGO densely. The abundant decoration of Au NRs not only enlarged the surface area of the composite and also could greatly quench ECL signal through effect of energy transfer efficiency (ECL-ET) due to the overlapped spectrum between the ECL spectrum and Au NRs’s absorption spectrum. Energy-dispersive analysis of X-rays (EDX) was further used to characterize the element component of the as-synthesized H-RGO-Au NRs (Fig. 2D).

**Figure 3:**
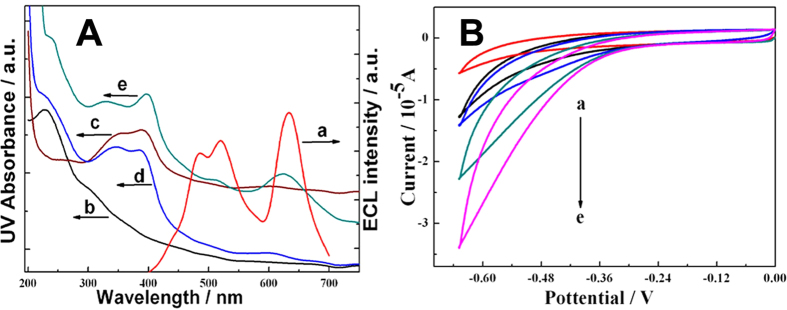
(**A**) ECL spectrum of the CdS:Eu QDs (a),and the UV-vis absorption spectra of GO (b), Hemin (c), H-RGO (d) and H-RGO-AuNRs (e); (**B**) Cyclic voltammograms of 3 mm H_2_O_2_ with addition of (b) hemin; (c) GO; (d) H-RGO, and (e) H-RGO-Au NRs with the same concentration in 3 mm H_2_O_2_ on GCE. Curve a is the CV response of 3 mm H_2_O_2_ without addition of any material.

UV-vis absorption spectra was also employed on water-dissolved GO, hemin, H-RGO and H-RGO-Au NRs respectively and the results were shown in Fig. 3A. From carve b, it can be clearly seen that GO exhibits its obvious absorption peak at 233 nm and shoulder peak at 300 nm corresponding to π–π* transitions of the aromatic C=C band and n–π* transitions of the C=O band, respectively. Besides, carve c shows that hemin has an obvious absorption peak at 388 nm which is attributed to the Soret band of hemin. Also another group of weak peaks between 500 and 700 nm ascribed to the Q-bands of hemin. However, after hemin was added on GO, the 388 nm peak red shifted to 413 nm (carve d), which was deemed to the reason of adsorption of hemin molecule on GO driven by π–π stacking interactions between the porphyrin moiety and RGO. After continuous addition of HAuCl_4_ and corresponding reduction the appearance of two new absorption peaks respectively at approximately 520 and 620 nm were consistent with the surface plasmon absorption of Au NRs. And this phenomenon indicated the generation of H-RGO-Au NRs complex.

For the application of ECL-RET in biosensor, the most important point is to choose an effective ECL donor and a suitable acceptor, with an essential condition that the donor’s ECL spectrum should be overlapped with the acceptor’s absorption spectrum. As shown in carve a of Fig. 3A, two spectral bands could be in the ECL emission spectrum of the CdS:Eu film: the one which was from 450 to 550 nm came out of the host CdS, and the other one from 600 to 700 nm was attributed to the energy transfer from host CdS to Eu^3+^ ions^32^. Exhilaratingly, from carve e of Fig. 3A, we can see that the Au NRs from the prepared ternary complex have two absorption peaks which completely overlap the ECL emission spectrum of CdS:Eu film. This great spectrum overlap between donor and acceptor could generate higher energy transfer efficiency in the ECL-ET system and was expected to show excellent performance in the construction of ECL-biosensor.

Cyclic voltammograms (CVs) were first used for catalytic activity characterization of different materials under the potential between −0.65 to 0 V (100 mV s^−1^). The current response at −0.65 V was used as the analytical signal. From Fig. 3B, we could find that hemin and GO showed catalytic activity in 3 mM H_2_O_2_ (curve b and c). However, H-RGO-Au NRs showed higher catalytic activity even compared with H-RGO (curve d and e). This phenomenon may be due to Au NRs attached on H-RGO-Au NRs composite which greatly improves the electron-transfer rate of graphene. The results demonstrated that the new composite H-RGO-Au NRs hold excellent catalytic ability for H_2_O_2_ attributed to the attached positively charged Au NRs on the surface of graphene which greatly improves its catalytic activity.

### Electrocatalytic reactivity mechanism of ECL biosensor

**Figure 4:**
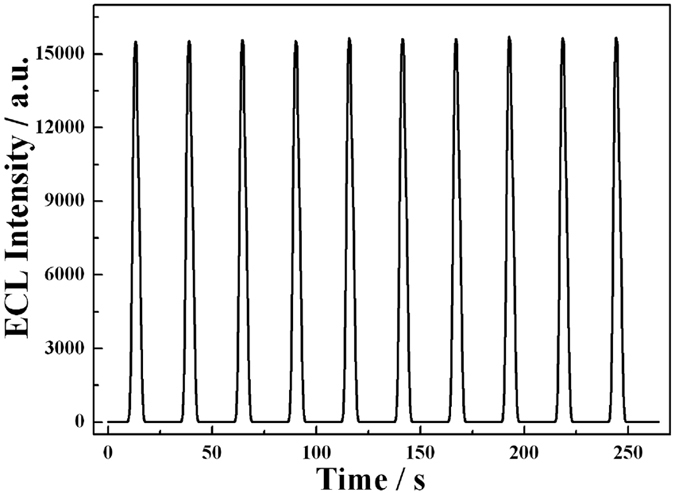
ECL emission from CdS:Eu QDs film on GCE in 7.5 mM H_2_O_2_ + 0.1 M KCl + 0.1 M PBS (pH = 7.4) under continuous cyclic potential scan for 10 cycles. The PMT voltage: −600 V. Potential: −1.3 V (vs Ag/AgCl). Scan rate, 100 mV/s.

In our design, glassy carbon electrode (GCE) was decorated by drop-coating of 10 μL CdS:Eu QDs which was used as ECL emitter. During the cathodic potential scan, the CdS:Eu QDs were reduced to CdS:Eu^−•^, and the coreactant H_2_O_2_ could react with CdS:Eu^−•^ to obtain an excited state (CdS:Eu*). This state could produce stable and high ECL signal in the aqueous solution and emit light at the same time (Fig. 4). The mechanism of this ECL process was listed as Eqs (1–3)^34^. Upon the addition of Hemin-RGO-Au, the ECL intensity decreased due to the occurred electrochemical reduction of hemin-RGO-Au. The reduced hemin-RGO-Au then chemically reduced the H_2_O_2_ and resulted to the efficient consumption of the coreactant. The process could be described by the following mechanism (Eqs (4) and (5))^30^,^35^.


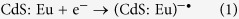



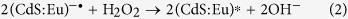



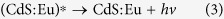



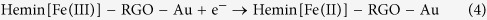



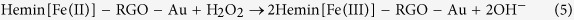


### The optimization of experimental conditions

**Figure 5:**
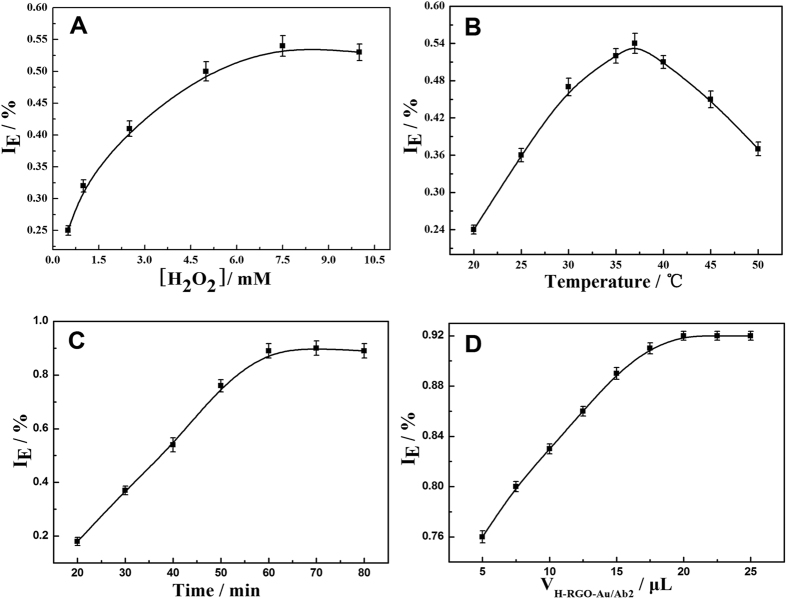
Effects of concentrations on quenching efficiency: optimization of H_2_O_2_ concentrations (**A**), CEA incubation temperature on GCE-CdS:Eu QDs/Ab_1_ (**B**), CEA incubation time on GCE-CdS:Eu QDs/Ab_1_ (**C**) and volumes of H-RGO-AuNRs/Ab_2_ in the ECL biosensor (**D**). The ECL peak intensity for analysis was obtained at −1.3 V (vs. Ag/AgCl) in 0.1 M PBS (pH 7.4), CV Scan rate: 0.1 V/s, the PMT voltage: −600 V.

The quenching efficiency of I_E_ = 1 − I/I_0_ (I, I_0_ are the ECL intensity treated with or without CEA respectively.) is used to study the effect of experimental conditions on ECL intensity. The experimental conditions mostly containing the concentration of H_2_O_2_, the temperature and time of Ab-CEA incubation, the volume of H-RGO-AuNRs/Ab_2_ tags. The concentration of H_2_O_2_ is important to the detection of biosensor. As can be seen in Fig. 5A, 7.5 mM H_2_O_2_ is the best concentration. Temperature of CEA incubated with GCE-CdS:Eu QDs/Ab_1_ are an important factor of the incubation efficiency which could also affect the further incubation between CEA and H-RGO-Au/Ab_2_. Figure 5B shows that the ECL intensity increase with the increasing temperature and reach the maximum at 37 °C which means 37 °C could be chosen as incubation temperature. Also, the incubation time is studied and shown in Fig. 5C, the ECL intensity associated with the time increase until 60 min, and then ECL intensity keeps invariant after 60 min. The concentration of CEA is 1.0 × 10^−10^ mg/mL during detection. Besides, Fig. 5D shows the volume of H-RGO-AuNRs/Ab_2_ tags could also influence the ECL intensity. The ECL intensity reach maximum when the volume increased to 20 μL, however, the signal remain unchanged when the volume continue to increase. So, 20 μL H-RGO-AuNRs/Ab_2_ tags is the optimized condition in this experiment.

### Characteristic of biosensing platform

**Figure 6:**
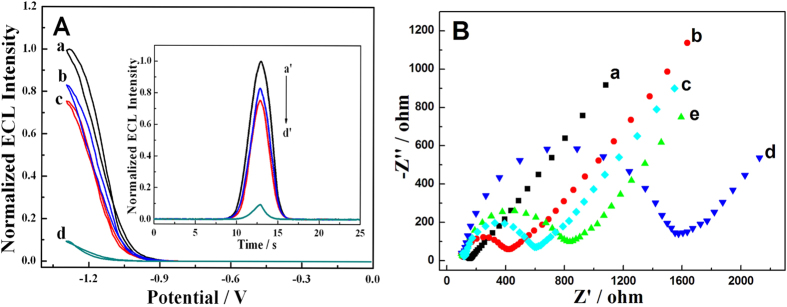
(**A**) The normalized ECL-potential curves of (a) GCE-CdS:Eu QDs, (b) GCE-CdS:Eu QDs/Ab_1_, (c) GCE-CdS:Eu QDs/Ab_1_/CEA and (d) GCE-CdS:Eu QDs/Ab_1_/CEA/Ab_2_/H-RGO-Au. Inset: the normalized ECL-time curves. The ECL peak intensity for analysis was obtained at −1.3 V (vs. Ag/AgCl) in 0.1 M PBS (pH 7.4) containing 7.5 mM H_2_O_2_. CV Scan rate: 0.1 V/s. The PMT voltage: 600 V. (**B**) Electrochemical impedance spectra of the proposed biosensing platform: (a) bare GCE; (b) GCE-CdS:Eu QDs; (c) GCE-CdS:Eu QDs/Ab_1_; (d) GCE-CdS:Eu QDs/Ab_1_/CEA; (e) GCE-CdS:Eu QDs/Ab_1_/CEA/H-RGO-Au/Ab_2_ in 0.1 M KCl solution containing 5.0 mM K_3_[Fe(CN)_6_]/K_4_[Fe(CN)_6_]. The PMT voltage: −600 V. Potential: −1.3 V (vs Ag/AgCl), Scan rate: 100 mV/s.

ECL signals at each immobilization step were also recorded to characterize the fabrication process of the biosensing platform. Figure 6A are signal-potential and signal-time curve of GCE-CdS:Eu QDs after added a scanning potential from −1.3 V to 0 V, respectively. And we can see in curve a that the ECL intensity of GCE-CdS:Eu QDs is strong and table with H_2_O_2_ as coreactant when the potential of electrodes becomes sufficiently negative. After modified with Ab_1_ and CEA, the ECL intensity of GCE-CdS:Eu QDs decreased a bit (curve b and c) because of the steric-hinerance effect. However, the ECL intensity of GCE-CdS:Eu QDs decreased a lot (curve d) after modified with H-RGO-Au/Ab_2_. This phenomenon was supposed due to the double-quenching effects caused by hemin and Au NRs.

At the same time, every step of the fabrication process of this ECL biosensor was continuously monitored by electrochemical impedance spectroscopy (Fig. 6B). The bare electrode displayed an almost straight line (curve a), which was characteristic of a diffusion process. While CdS:Eu QDs were assembled on the electrode surface, the electron−transfer resistance (Ret) increased obviously (curve b), which indicated that the CdS:Eu QDs were immobilized on the electrode surface and decreased the electron-transfer efficiency. Ab_1_ and following incubation of CEA resulted in a larger Ret (curve c and d). And this is mainly due to the poor electroconduction of biomolecules which results in steric hindrance and then inhibits the interfacial charge transfer. However, after following conjugated nanoprobe of H-RGO-Au/Ab_2_ onto the surface of Ab_1_ through specific interaction, an obvious of Ret decreases was appeared, which indicates that the synthesized H-RGO-Au/Ab_2_ possessed high conductivity, good electron transfer efficiency and excellent biocompatibility (curve e).

### Analytical performance

**Figure 7:**
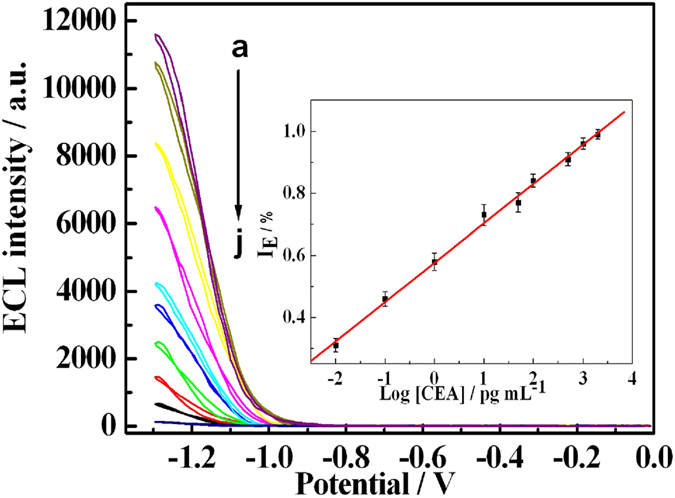
ECL signals response for detection of different concentrations of CEA. The concentrations of CEA: (a) 0, (b) 1.0 × 10^−14^ g/mL, (c) 1.0 × 10^−13^ g/mL, (d) 1.0 × 10^−12^ g/mL, (e) 1.0 × 10^−11^ g/mL, (f) 5.0 × 10^−11^ g/mL, (g) 1.0 × 10^−10^ g/mL, (h) 5.0 × 10^−10^ g/mL, (i) 1.0 × 10^−9^ g/mL and (j) 2.0 × 10^−9^ g/mL, inset: Linear relationship between quenching efficiency and the logarithm of CEA concentration, three measurements for each point. The PMT voltage: −600 V. Potential: −1.3 V (vs Ag/AgCl).

Under the optimization of experimental conditions, the sensitivity of the ECL biosensor based on CdS:Eu QDs and H-RGO-Au NRs were studied by changing the concentration of CEA. From Fig. 7, ECL intensity is found to be very sensitive and decreased with increased CEA concentration form 1.0 × 10^−14^ to 2.0 × 10^−9^ g/mL. Besides, the inset of Fig. 7 show that quenching efficiency (I_E_) was also logarithmic related to the CEA concentration with R = 0.998 and limit of detection (LOD) was 0. 01 pg/mL, which was comparable with that of 0.06 pg/mL CEA by hybrid gold/silica/CdSe-CdS quantum-dot nanostructure based ECL immunosensor^36^. And it much lower than that of Au-polydopamine functionalized carbon encapsulated Fe_3_O_4_ magnetic nanocomposites based biosensor (0.33 pg/mL CEA)^37^, polybead carried gold nanoparticles assistant biosensor (0.12 pg/mL CEA), electrochemical immunoassay with branched electrode system (0.05 ng/mL CEA)^38^, and photoelectrochemical immunoassay (2.1 pg/mL CEA)^39^ or fluorescent sensors (5.0 pg/mL CEA)^40^. This low LOD was supposed to be the reasons as following: the strong and stable ECL intensity of CdS:Eu QDs, the high electro-catalysis effect of H-RGO-Au NRs to coreactant and the energy transfer quenching effect of Au NRs.

### The selectivity, repeatability and stability of ECL biosensor

The practicality ECL biosensor was measured by the selectivity, repeatability and stability. The detection signal of 1.0 pg/mL CEA, 50 pg/mL AFP, 50 pg/mL PSA, 100 pg/mL BSA mixture was compared with pure CEA. And the result shows that the signal caused by mixture has no obvious difference with the signal caused the pure CEA, and the relative standard deviation between them is 7.26% which means the designed ECL biosensor has an excellent selectivity to detect CEA. Meanwhile, the repeatability was also been studied. 1.0 pg/mL CEA solution was detected by the same biosensor for five times with the relative standard deviation of 3.28%. Besides, 1.0 pg/mL CEA solution was also detected business five electrodes and got a relative standard deviation of 5.40%. Those result indicated the designed ECL biosensor has a favorable repeatability. Finally, the stability of the biosensor was studied. The biosensor has been kept in pH = 7.4 PBS buffer at 4 °C for a week before detection. And the ECL response of treated electrode is almost the same as the electrode without be treated which means the nice stability of the biosensor.

### Application in analysis of samples

The practical application of the immunoassay was investigated by analyzing the recoveries of different concentrations of CEA in human serum samples. In order to confirm the practicality and reliability of this ECL biosensor in clinical application, the standard addition method was used to detect the content of CEA in human serum. The result shows that the recovery of the ECL biosensor was between 97.5–110% which indicates this designed biosensor has a potential to be used in CEA clinical detection (Table 1).

## Conclusion

In summary, a novel ECL biosensor for ultrasensitive CEA detection was built based on the high electrocatalytic activity of ternary complexes for the reduction of H_2_O_2_, acted as the coreactant of QDs-based ECL, and the ECL-RET strategy between the large amount of Au NRs on the graphene triple complex surface and the CdS:Eu QDs. This simple and ultrasensitive ECL biosensor combined the advantage of the high electrocatalytic activity of H-RGO-Au NRs and the superiority ECL-RET strategy between the Au NRs and the CdS:Eu QD. Besides, a dynamic detection for monitoring the concentration of CEA was also achieved in this biosensor based on the electrochemical method. The designed biosensor showing simple operation, wide linearity range, high sensitivity and excellent selectivity, provided a new approach for ECL immunoassay sensing and significant prospect for practical application.

## Materials and Methods

### Chemicals and reagents

Poly (sodium 4-styrenesuifonate) solution (PSS) was obtained from Sigma-Aldrich. Carcinoembryonic antigen (CEA) was purchased from Zhenglong Biochem.Lab and used without further purification. Graphene oxide was gotten from Nanjing XFNANO Materials Tech Co., Ltd. Cadmium nitrate tetrahydrate (Cd(NO_3_)_2_), Europium(III) nitrate hexahydrate (Eu(NO_3_)_3_), 3-Mercaptopropionic acid (MPA), N-Hydroxysuccinimide (NHS), N-(3-Dlmethylamlnopropyj)-N’-ethylcarbodll mide hydrochlorlde (EDC), Bovine serum albumin (BSA), Chloroauric acid (HAuCl_4_) were obtained from Aladdin. Hematin Chloride was purchased from Shanghai Generay Biotech Co., Ltd. All aqueous solutions were prepared using ultra-pure water (Milli-Q, Millipore).

### Apparatus

All ECL measurements were carried on a MPI-A multifunctional electrochemical and chemiluminescent analytical system (Remax Electronic Instrument Limited Co., Xi’an, China). A three-electrode configuration was used in electrochemical and ECL experiments with a glassy carbon electrode (GCE, 3 mm diameter) as the working electrode, Pt wire served as the counter electrode, and Ag/AgCl electrode with saturated KCl solution as reference electrode, respectively. The ECL emission measurements were conducted in 0.1 M pH 7.4 PBS using H_2_O_2_ as a coreactant. The emission window was placed in front of the photomultiplier tube (PMT) biased at −600 V.

### Synthesis of CdS:Eu QDs

The CdS:Eu QDs were prepared as our previous report^34^,^35^. 0.08 M Eu (NO_3_)_3_ solution (112.5 μL) was added to 30 mL of aqueous solution containing 0.1683 g Cd(NO_3_)_2_·4H_2_O under stirring and heated to 70 °C. Then, a freshly prepared Na_2_S·9H_2_O_2_ solution (0.7205 g in 30 mL of ultrapure water) was injected to obtain orange-yellow precipitates. This reaction stayed at 70 °C for 3 h with continuous refluxing. The obtained final reaction precipitates were centrifuged and washed thoroughly with absolute ethanol and for ultrapure water three times to get rid of the remaining Eu^3+^ and other ions. The finally precipitate was ultrasonically dispersed into water and then the upper yellow solution of CdS:Eu QDs were collected after centrifugation. The final solution could be rather stable for 1 month when stored in a refrigerator at 4 °C.

### Preparation of CdS:Eu QDs Film

The GCE was polished with alumina, sonicated in ethanol and ultrapure water and then thoroughly rinsed with water. To prepare the CdS:Eu QDs film, 10 μL of the CdS:Eu QDs solution was dropped onto the pretreated GCE surface at room temperature. Then, the CdS:Eu NC-modified GCE was stored in 0.1 M NaCl + 0.1 M PBS (pH 7.4) for further use.

### Construction of ECL Biosensor

The CdS:Eu QDs modified GCE was treated with 1.5 mM MPA for 3 h at 4 °C, then the terminal carboxylic acid groups of the MPA on GCE surface were activated using 20 mg of EDC and 10 mg of NHS which were dispersed in 1.0 mL of 0.1 M imidazole-HCl buffer (pH 6.8) for 1 h at room temperature. After rising the electrodes with 0.1 M PBS (pH 7.4), 100 μg mL^−1^ Ab_1_ solution was introduced to the electrode and incubated at 4 °C for 12 h. Following this, the above electrode was immersed in 2 wt% BSA solution for 40 min to block the nonspecific active binding sites. Finally, the electrode (marked as GCE/CdS:Eu QDs/Ab_1_) was washed by PBS and stored in 4 °C when not used.

### Preparation of Hemin-Reduced Graphene Oxide (H-RGO)

Hemin-reduced graphene oxide (H-RGO) was synthesized according to our previous reported procedure^41^. First, 5 mL GO (2 mg/mL) was dispersed in 35 mL H_2_O in a 100 mL dried three-necked flask. After stirring and ultrasonication for 30 min, ammonia solution (60 μL) and hemin (10 mg) were added to the mixture, followed by the addition of hydrazine solution (10 μL). The solution was heated in a water bath at 60 °C for 4 h. The final product was collected after 10000 rpm centrifugation for 25 min and then washed and dried in vacuo for further use.

### Preparation of H-RGO-Au NRs

According to the provious work^30^, the seed solution was first prepared for Au NRs. 1 mL 0.12 mM HAuCl_4_ was mixed with 1 mL 0.048 mM CTAB solution. Then fresh prepared NaBH_4_ (2.4 mM, 0.12 mL) was added into above Au (III)-CTAB solution under vigorous stirring for 2 min. until the solution color changed from yellow to brownish-yellow. The prepared seed solution was aged at 27 °C for 2 h before use. Then the growth solution was prepared using 50 mL 0.2 mM HAuCl_4_ together with 50 mL 0.048 M CTAB, 2.5 mL of 0.96 mM AgNO_3_ and 3.4 mg H-RGO product under gently mixing at room temperature. Then 0.7 mL 0.02 M AA was added in to above mixture. The color of the solution could be found to fade to colorless from dark-yellow. Finally, 0.12 mL prepared seed solution was gently added into this solution at 27 °C for 20 min and then was kept at 30 °C for 12 h for NR growth on the surface of H-RGO. The final products were collected by centrifugation at 8500 rpm for 25 min and were then re-dispersed in water.

### Preparation of H-RGO-Au NRs/Ab_2_

The Ab_2_-labeled H-RGO-Au NRs were prepared as follows: H-RGO-Au NRs (0.1 mg mL^−1^, 0.5 mL) were mixed with PSS solution (200 μL, 30%), ultrasonic for 5 min, and vibrated for 2 h, and then centrifuged to remove the excess PSS, and scattered in 400 μL PBS. Subsequently, a solution of Ab_2_ (50 μg L^−1^, 100 μL) was added. The mixture was gently stirred at room temperature for 1 h and then centrifuged. After washing with PBS, the pellet was resuspended in PBS (500 μL) containing 2% BSA and stored at 4 °C for further use.

### ECL Measurements

Different concentrations of CEA (20 μL) were dropped onto the surface of the modified electrode and incubated at 37 °C for 40 min. Subsequently, the electrode was washed and treated with 20 μL H-RGO-Au NRs/Ab_2_ solution for 60 min incubation at 37 °C. The following ECL measurement was conducted in 0.1 M PBS (pH 7.4) containing 0.10 M KCl, 7.5 mM H_2_O_2_ and scanned from 0 to −1.3 V after washing the electrode to remove the unbound H-RGO-Au NRs/Ab_2_ bioconjugates. The voltage of the PMT was set at −600 V in the process of detection.

## Additional Information

**How to cite this article**: Liu, J. *et al*. Efficient double-quenching of electrochemiluminescence from CdS:Eu QDs by hemin-graphene-Au nanorods ternary composite for ultrasensitive immunoassay. *Sci. Rep.* **6**, 30577; doi: 10.1038/srep30577 (2016).

## Figures and Tables

**Figure 1:**
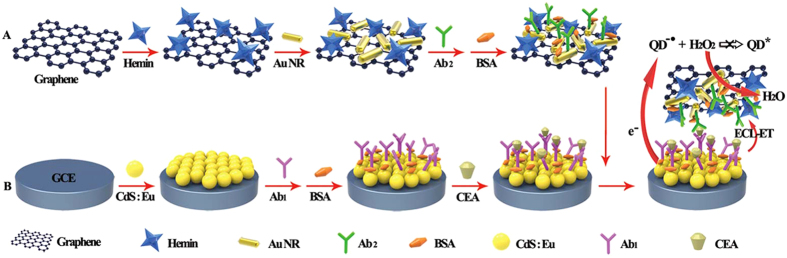
Schematic illustration of the fabrication process the CEA biosensing platform based on double-quenching of H-RGO-AuNRs. Process (**A,B**) respectively represents the preparation of hemin-reduced graphene oxide-Au nanorods (H-RGO-AuNRs) and CEA biosensing platform.

**Table 1:** The result of CEA determination in real human serum by the immunosensor.

Sample	Standard CEA value (pg/mL)	Found (pg/mL)	Recovery (%)
1	0.07	0.061	105
2	0.7	0.72	97.5
3	7	6.596	102
4	70	70.417	110
5	700	660.036	108
